# Mettl14-mediated m6A modification modulates neuron apoptosis during the repair of spinal cord injury by regulating the transformation from pri‐mir‐375 to miR-375

**DOI:** 10.1186/s13578-020-00526-9

**Published:** 2021-03-11

**Authors:** Haoyu Wang, Jing Yuan, Xiaoqian Dang, Zhibin Shi, Wenrui Ban, Dong Ma

**Affiliations:** 1 Department of Orthopedics, Xi’an Jiaotong University Second Affiliated Hospital, Xi’an, 710004 Shanxi People’s Republic of China; 2 Xi’an Radio and Television University, Xi’an, 710002 Shanxi People’s Republic of China; 3 Key Laboratory of Shanxi Province for Craniofacial Precision Medicine Research, College of Stomatology, Xi’an Jiaotong University, 98 XiWu Road, Xi’an, 710004 Shaanxi China

**Keywords:** Spinal cord injury, m6A modification, Mettl14, RASD1, miR-375, Pri‐mir‐375

## Abstract

**Background:**

Spinal cord injury (SCI) is a disabling disorder, resulting in neurological impairments. This study investigated the mechanism of methyltransferase-like 14 (Mettl14) on apoptosis of spinal cord neurons during SCI repair by mediating pri-microRNA (miR) dependent N6-methyladenosine (m6A) methylation.

**Methods:**

The m6A content in total RNA and Mettl14 levels in spinal cord tissues of SCI rats were detected. Mettl14 expression was intervened in SCI rats to examine motor function, neuron apoptosis, and recovery of neurites. The cell model of SCI was established and intervened with Mettl14. miR-375, related to SCI and positively related to Mettl14, was screened out. The expression of miR-375 and pri-miR-375 after Mettl14 intervention was detected. The expression of pri-miR-375 combined with DiGeorge critical region 8 (DGCR8) and that modified by m6A was detected. Furthermore, the possible downstream gene and pathway of miR-375 were analysed. SCI cell model with Mettl14 intervention was combined with Ras-related dexamethasone-induced 1 (RASD1)/miR-375 intervention to observe the apoptosis.

**Results:**

Mettl14 level and m6A content in spinal cord tissue were significantly increased. After Mettl14 knockdown, the injured motor function was restored and neuron apoptosis was reduced. In vitro, Mettl14 silencing reduced the apoptosis of SCI cells; miR-375 was reduced and pri-miR-375 was increased; miR-375 targeted RASD1. Silencing Mettl14 inactivated the mTOR pathway. The apoptosis in cells treated with silencing Mettl14 + RASD1/miR-375 was inhibited.

**Conclusions:**

Mettl14-mediated m6A modification inhibited RASD1 and induced the apoptosis of spinal cord neurons in SCI by promoting the transformation of pri-miR-375 to mature miR-375.

## Background

Spinal cord injury (SCI) is a severe central nervous system injury, which leads to the loss of motor and sensory functions in SCI patients and seriously affects physical and mental health [1, 2]. As a sudden life-changing event, SCI results in neuromuscular weakness that impacts respiratory function, and requires complex and long-term rehabilitation [3, 4]. The common traumatic causes for SCI are traffic and sports accidents and gun-shot wound, while infections and vascular dysfunction are contributors to non-traumatic SCI [5]. More than one million patients around the world are paralyzed by SCI, which not only brings serious social and economic problems to patients and their families, but also to society [6]. Neuronal apoptosis is a key pathological feature of SCI [7]. SCI causes increased apoptosis of neurons, resulting in irreversible spinal cord dysfunction [8]. Therefore, it is very crucial to develop a new therapy for SCI in terms of neuronal apoptosis.

RNA modification, as an early adaptation mechanism of cells to environmental changes, has become an important target of tumour therapies and a hot spot of epigenetic research [9]. Post-transcriptional modifications affect cellular RNA stability, and the commonly seen modification is N6‑methyladenosine (m6A) [10, 11]. Aberrant m6A is related to the progression of diseases and cancers [12]. The methyltransferase-like 14 (Mettl14) is one of the several methyltransferases critical for mediating m6A modification [13, 14]. For example, Mettl14 positively modulates m6A modification in breast cancer cells [12]. However, the role of Mettl14-mediated m6A modification in SCI is less studied. Mettl14 prevents the metastasis of hepatocellular carcinoma via m6A-dependent primary microRNA (miR) processing [15]. miRs are important factors to coordinate key molecular pathways in SCI, which can serve as an organized network to control apoptosis, inflammation, oligodendrocyte development and axon regeneration after SCI [16, 17]. Based on the above information, we hypothesize an underlying mechanism of Mettl14 in SCI via m6A-dependent miR processing. Therefore, the comprehensive experiments were conducted to testify the involvement of Mettl14 and m6A-dependent miR in SCI models, which may offer novel insights for therapeutic interventions for SCI patients.

## Results

### Mettl14-mediated m6A modification was involved in SCI

**Fig. 1:**
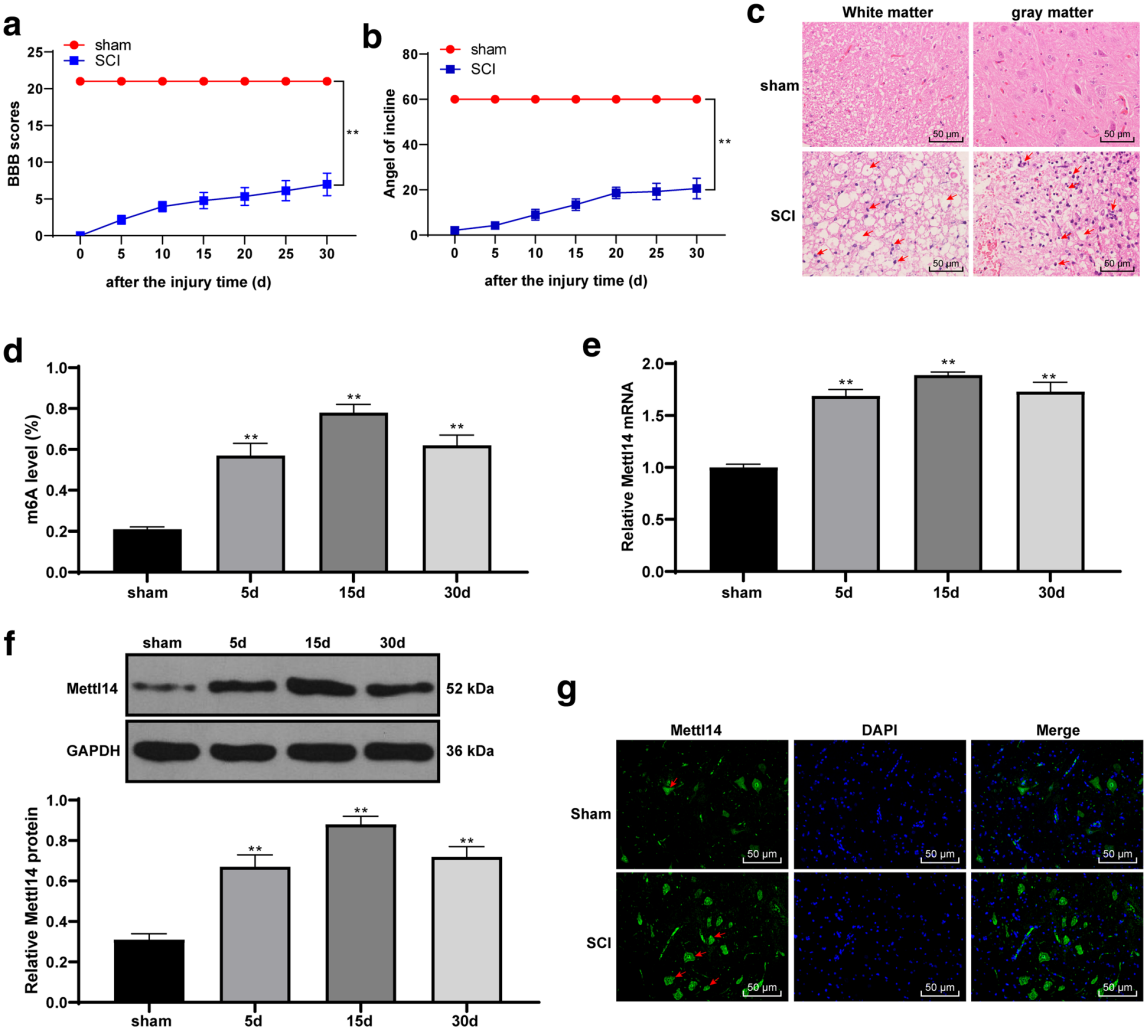
Mettl14 and m6A were significantly upregulated after SCI. After the SCI model was established, the motor function of rats in each group was assessed by BBB motor function score (**a**) and bevel test score (**b**) on the 0, 5, 10, 15, 20, 25 and 30 days. The SCI at 2-cm transverse section of the spinal cord centred on the injured site was assessed by HE staining (**c**) on the 30th day; the neurons at the point of arrow were obviously lost; and the m6A content in total RNA of rats at the 5th day, 10th day and 30th day after SCI was assessed by EpiQuik m6A RNA Methylation quantification kit (**d**). The expression of Mettl14 at the 5th day, 10th day and 30th day after SCI was detected by RT-qPCR (**e**) and Western blot (**f**); and the fluorescence intensity of Mettl14 in a 2-cm transverse section of the spinal cord centred on the injured site on the 30th day was detected using immunofluorescence (**g**), and the arrow indicates Mettl14 positive expression. N = 8. Each experiment was repeated three times independently, and the data are expressed as mean ± standard deviation. The data in panels **a**, **b** were analysed by two-way ANOVA, and data in panels **d**–**f** were analysed by one-way ANOVA, followed by Tukey’s multiple comparisons test. Compared with the sham group, *p < 0.05, **p < 0.01

As the most abundant methylation modification on RNA, m6A involves many steps in the process of mRNA processing, so as to regulate gene expression. But the role of m6A modification in SCI has not been elucidated. To explore this, we first established a SCI rat model, and used Basso, Beattie and Bresnahan (BBB) motor function scores and bevel test score to evaluate SCI in rats. The results showed that after SCI, rats immediately developed paralysis of hind limbs, and the motor function of rats partially recovered on the 5th day after SCI treatment (Fig. 1a, b). The spinal cord tissue of rats on the 30th day was taken, and then HE staining was adopted to evaluate the SCI in rats. After SCI, the central gray matter and peripheral white matter of rats were destroyed, and the motor neurons of anterior horn were significantly lost (indicated by the arrow) (Fig. 1c). Epiquik m6A RNA measurement quantification kit was used to detect the content of m6A in the spinal cord of rats in different time points. The content of m6A in total RNA of SCI rats was significantly increased (Fig. 1d). The literature pointed out that the core m6A writers are Mettl3 and Mettl14, which both contain methylransferase domains [18]. Mettl14 promotes breast cancer development by regulating m6A and hsa‑miR‑146a‑5p [12]. Therefore, we wondered whether Mettl14 can influence the development of SCI by regulating m6A. Then Mettl14 expression was detected by RT-qPCR, Western blot and immunofluorescence. Mettl14 levels were significantly increased in the spinal cord of SCI rats at the 30th day, and fluorescence intensity was enhanced (Fig. 1e–g) (all *p* < 0.01). It can be seen that Mettl14 and m6A were significantly upregulated after SCI.

### ShRNA-Mettl14 reduced SCI and promoted the recovery of neurites after SCI

**Fig. 2:**
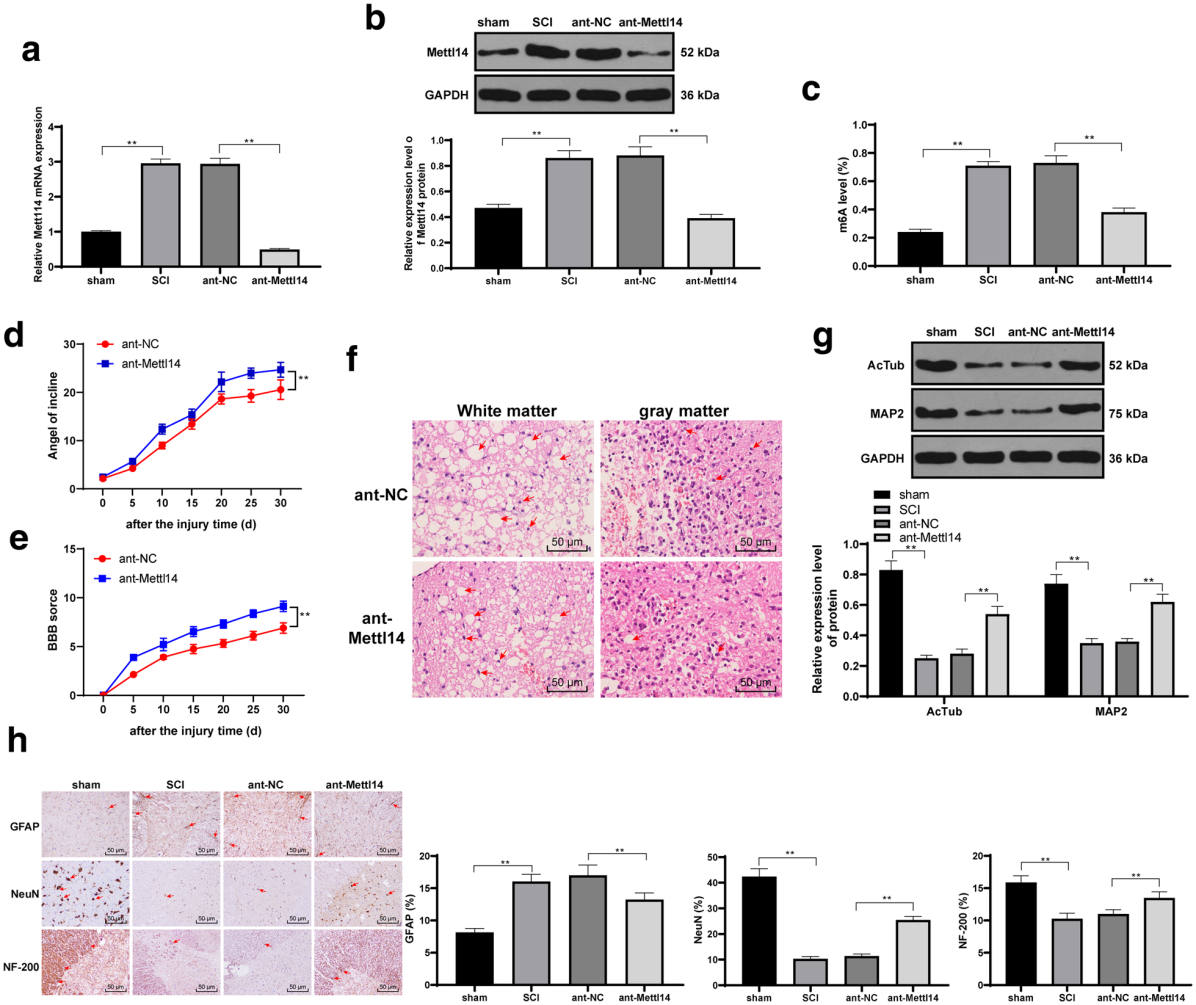
ShRNA-Mettl14 can relieve SCI and promote the recovery of neurites after SCI. ShRNA-Mettl14 was injected into SCI model rats using lumbar puncture via the tail vein. The equal distance transverse section on 2-cm spinal cord centred on the injured site was made. Mettl14 levels in the spinal cord tissue of rats at the 30th day were detected by RT-qPCR (**a**) and Western blot analysis (**b**). At the 30th day after SCI, the content of m6A in the total RNA of the spinal cord was measured by EpiQuik m6A RNA methylation quantification kit (**c**). BBB motor function score (**e**) and level test score (**d**) were used to evaluate the motor function of the rats in each group. The rats were euthanized at 30th day, and HE staining was performed (**f**); the arrow shows the weakened destruction of central gray matter and peripheral white matter; Western blot analysis (**g**) were used to measure the expression of AcTub and MAP2. The positive expression of GFAP, NeuN and NF-200 was evaluated by immunohistochemical staining; and the arrow indicates GFAP, NeuN and NF-200 positive expression (**h**). N = 8. Each experiment was repeated three times independently. The data are expressed by mean ± standard deviation. The data in panels A/B/C/G were analysed by one-way ANOVA, and data in panels **d–f** were analysed by two-way ANOVA, followed by Tukey’s multiple comparisons test. *p < 0.05, **p < 0.01

To confirm the effect of Mettl14 on SCI, we injected shRNA-Mettl14 into rats using lumbar puncture and successfully reduced Mettl14 expression (Fig. 2a, b) (*p* < 0.01). The content of m6A in the total RNA of the spinal cord was measured. It showed the content of m6A in the total RNA of the spinal cord decreased significantly after shRNA-Mettl14 injection (Fig. 2c) (*p* < 0.01). BBB and Bevel test score evaluated the motor function of rats, and showed that the motor function of rats was restored after shRNA-Mettl14 injection (Fig. 2d, e) (*p* < 0.01). The rats were euthanized on the 30th day. HE staining revealed that the damage of central gray matter and peripheral white matter was weakened after shRNA-Mettl14 injection (Fig. 2f). Microtubule associated protein 2 (MAP2) is a specific structural protein in neurons, which is mainly expressed in the dendrites of neurons and has the function of stabilizing microtubules and regulating the length of dendrites. Therefore, we detected the expression of AcTub and MAP2. After SCI treatment, the expression of AcTub and MAP2 in the spinal cord of rats decreased significantly, while after ant-Mettl14 injection, AcTub and MAP2 increased significantly (all *p* < 0.05) (Fig. 2g), suggesting that shRNA-Mettl14 is helpful to stabilize microtubule structure and repair neurites. Axonal regeneration is beneficial to the recovery of motor function of hind limbs in rats with spinal cord defect, and the scar caused by astrocytes at the edge of injury will hinder axonal regeneration; NeuN, GFAP and NF-200 are used to evaluate neuronal, axonal regeneration and glial scab formation in the spinal cord [19, 20]. Then, to determine whether shRNA-Mettl14 affects the repair of spinal cord defect, we detected the positive expression of GFAP, NeuN and NF-200 by immunohistochemistry. After shRNA-Mettl14 injection, the expressions of GFAP and NF-200 were decreased, while that of NeuN was increased significantly (all *p* < 0.05) (Fig. 2h). In conclusion, shRNA-Mettl14 can relieve SCI and promote the recovery of neurites after SCI.

### ShRNA-Mettl14 inhibited apoptosis of spinal neurons in SCI rats

**Fig. 3:**
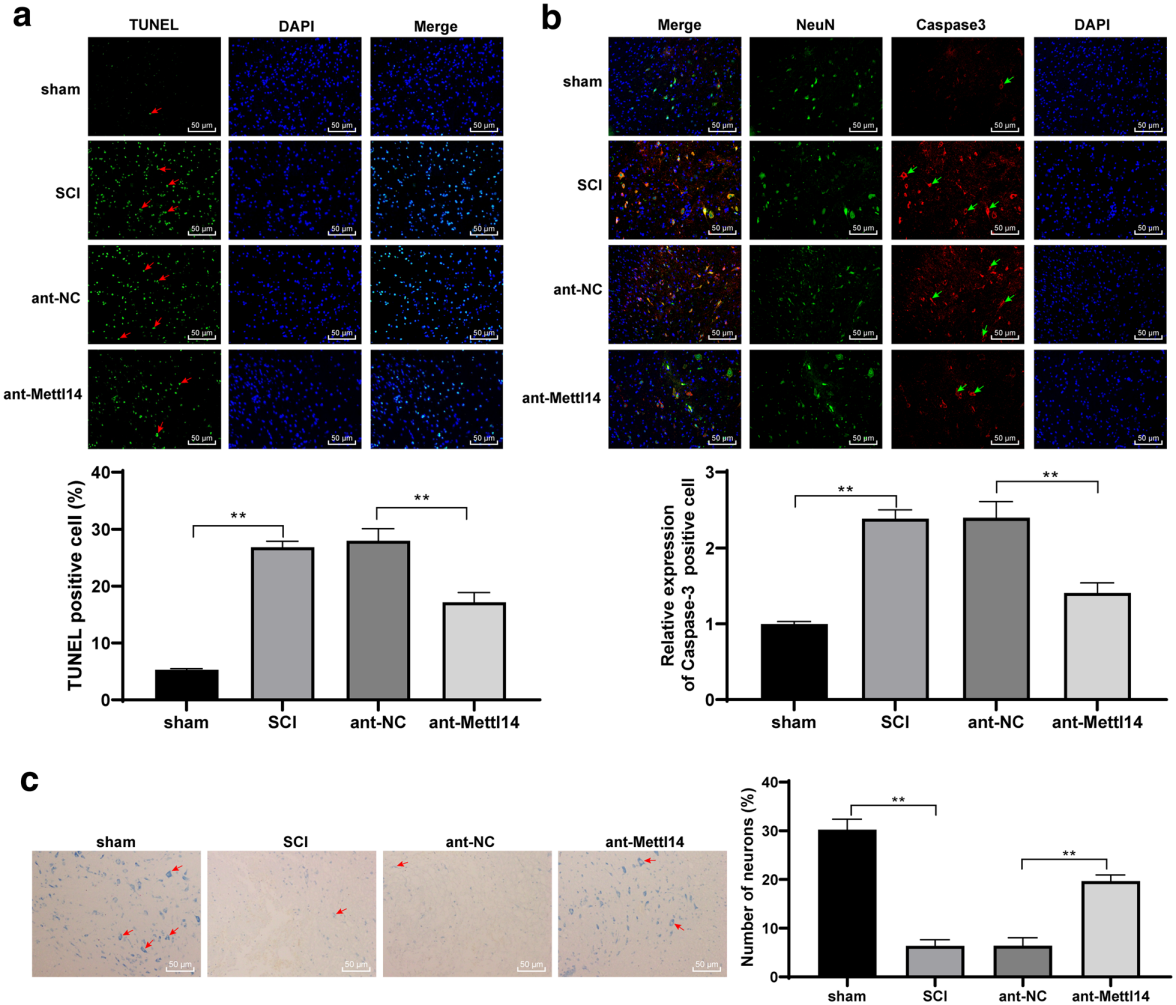
ShRNA-Mettl14 inhibited apoptosis of spinal neurons in SCI rats. ShRNA-Mettl14 was injected into SCI model rats using lumbar puncture via the caudal vein. The equal distance transverse sections on 2-cm spinal cord centred on the injured site were made at the 30th day. The apoptosis of spinal cord cells was detected by **a** TUNEL and **b** immunofluorescence staining, and the survival number of neurons was observed by **c** Nissl staining; the arrows indicate TUNEL-positive cells, NeuN + caspase-3 positive cells or Nissl stained neurons. N = 8. Each experiment was repeated three times independently. The data are expressed by mean ± standard deviation. The data in panels **a**–**c** were analysed by one-way ANOVA, followed by Tukey’s multiple comparisons test. *p < 0.05, **p < 0.01

Apoptosis plays an important role in the repair of SCI [21]. Then, to explore whether Mettl14 expression also affects the apoptosis of cells in SCI, we detected the apoptosis of neurons in spinal cord tissues of rats at the 30th day, and caspase-3 expression. After shRNA-Mettl14 injection, TUNEL-positive cells and NeuN + caspase-3-positive cells were significantly decreased (*p* < 0.01) (Fig. 3a, b). The survived neurons were observed by Nissl staining, and it revealed that the neurons survived in the spinal cord of SCI rats were increased significantly after shRNA-Mettl14 injection (*p* < 0.01) (Fig. 3c).

### Mettl14 knockdown inhibited H_2_O_2_-induced apoptosis in SCI cell model

**Fig. 4:**
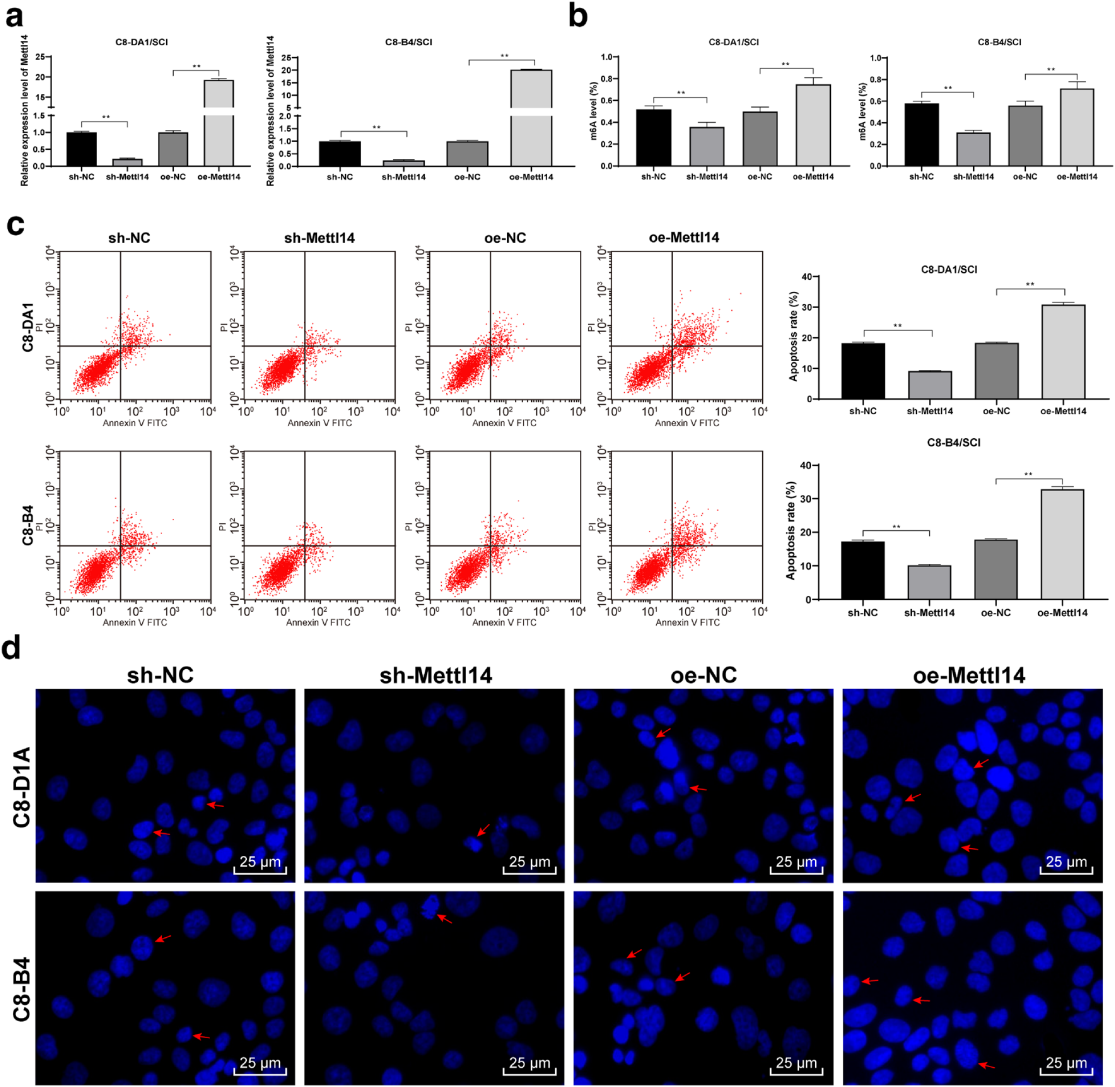
ShRNA-Mettl14 inhibited H2O2-induced apoptosis in SCI cell model. C8-DA1 and C8-B4 cells induced by H2O2 were used to establish the cell model of SCI in vitro. Mettl14 expression was detected by RT-qPCR (**a**). The content of m6A in the total RNA of the spinal cord was measured by EpiQuik m6A RNA methylation quantification kit (**b**). Then flow cytometry (**c**) and Hoechst staining (**d**) were used to evaluate the apoptosis. Each experiment was repeated three times independently. The data are expressed by mean ± standard deviation. The data in panels A/B/C were analysed by one-way ANOVA, followed by Tukey’s multiple comparisons test. *p < 0.05, **p < 0.01

To further explore the molecular mechanism of Mettl14 in apoptosis induced by SCI, we used H_2_O_2_ to induce glial cells (C8-DA1) and neurons (C8-B4) to simulate the SCI cell model, and intervened Mettl14 expression in cells, and then detected the change of Mettl14 expression and the change of m6A content in total RNA 24 h later. The knockdown and overexpression of Mettl14 was successfully transferred into C8-DA1 and C8-B4 cells (Fig. 4a), and the content of m6A was decreased significantly after Mettl14 knockdown, but increased significantly after overexpression of Mettl14 (all *p* < 0.01) (Fig. 4b). The apoptosis of C8-DA1 and C8-B4 cells was evaluated by flow cytometry and Hoechst staining. The treatment of Mettl14 knockdown significantly reduced the apoptosis of H_2_O_2_-induced C8-DA1 and C8-B4 cells, while overexpression of Mettl14 aggravated the apoptosis of C8-DA1 and C8-B4 cells (Fig. 4c, d). These results suggested that Mettl14 knockdown can reduce the apoptosis of C8-DA1 and C8-B4 cells induced by H_2_O_2_.

### Mettl14-mediated m6A modification promoted the transformation from pri‐mir‐375 to miR-375

**Fig. 5:**
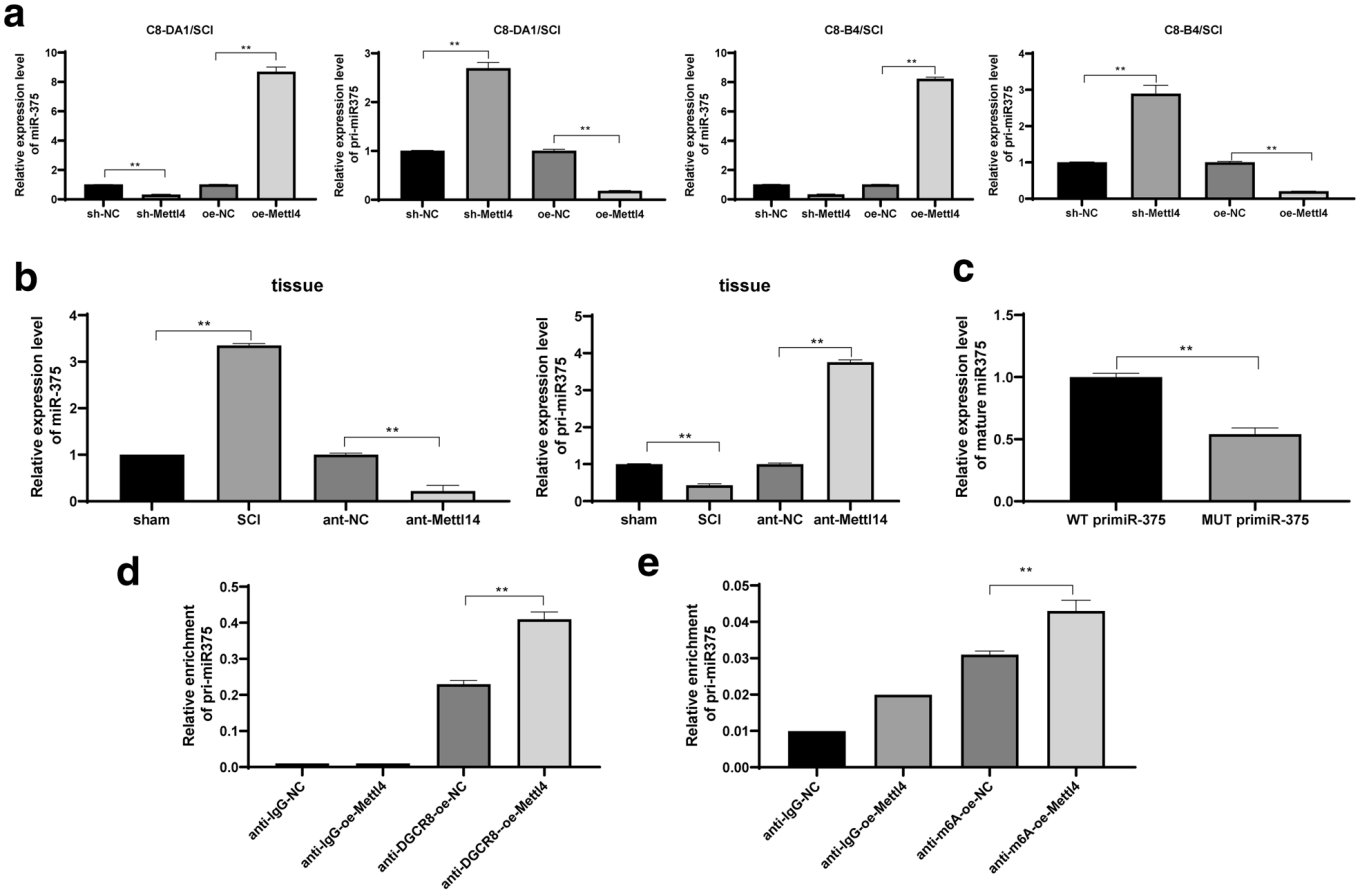
Mettl14-mediated m6A modification to promote the transformation from pri-miR-375 to miR-375. RT-qPCR was used to detect miR-375 and pri-miR-375 in cells after transfection for 24 h or spinal cord tissue of rats on the 30th day of each group (**a**, **b**), and then RT-qPCR was used to detect the effect of m6A mutation in pri-miR-375 on miR-375 processing (**c**); RIP assay was used to evaluate the effect of overexpression of Mettl14 on pri-miR-375 and miR-375 modified by m6A (**d**, **e**). Each experiment was repeated three times independently. The data are expressed by mean ± standard deviation. The data in panel B were analysed by the t test, and the data in panels **a**, **c**–**e** were analysed by one-way ANOVA, followed by Tukey’s multiple comparisons test. **p < 0.01

m6A can affect the progression of the disease by affecting pri-miR transformation to mature miR [22], while miR-375 expression is upregulated after spinal cord contusion in rats, which has an effect on the recovery of spinal cord contusion function [23]. Therefore, we speculated that the modification of m6A mediated by Mettl14 plays a negative role by regulating miR-375 expression in SCI. First of all, we found that miR-375 in cells and tissues was decreased and pri-miR-375 was increased significantly after Mettl14 knockdown; while miR-375 in cells was increased significantly after overexpression of Mettl14, and pri-miR-375 accumulation was decreased in cells (Fig. 5a, b) (all *p* < 0.01). Then, RT-qPCR showed that the mutation of m6A site in pri-miR-375 was significantly reduced the processing of mature body (Fig. 5c). The DGCR8 can actively regulate the transformation of pri-miR to mature miR [22]. Therefore, we used DGCR8 to detect the expression of pri-miR-375 and miR-375 by immunocoprecipitation. We found that overexpression of Mettl14 promoted the combination of DGCR8 and pri-miR-375 (Fig. 5d). In addition, RIP assay unveiled that overexpression of Mettl14 clearly elevated the expression of m6A-modified pri-miR-375 (Fig. 5e). To sum up, the findings above showed that the decrease of Mettl14 expression results in the decrease of m6A level, thus the stability of pri-miR-375 reduced by m6A modification is restored, and the transformation from pri-miR-375 to miR-375 is blocked.

**Fig. 6:**
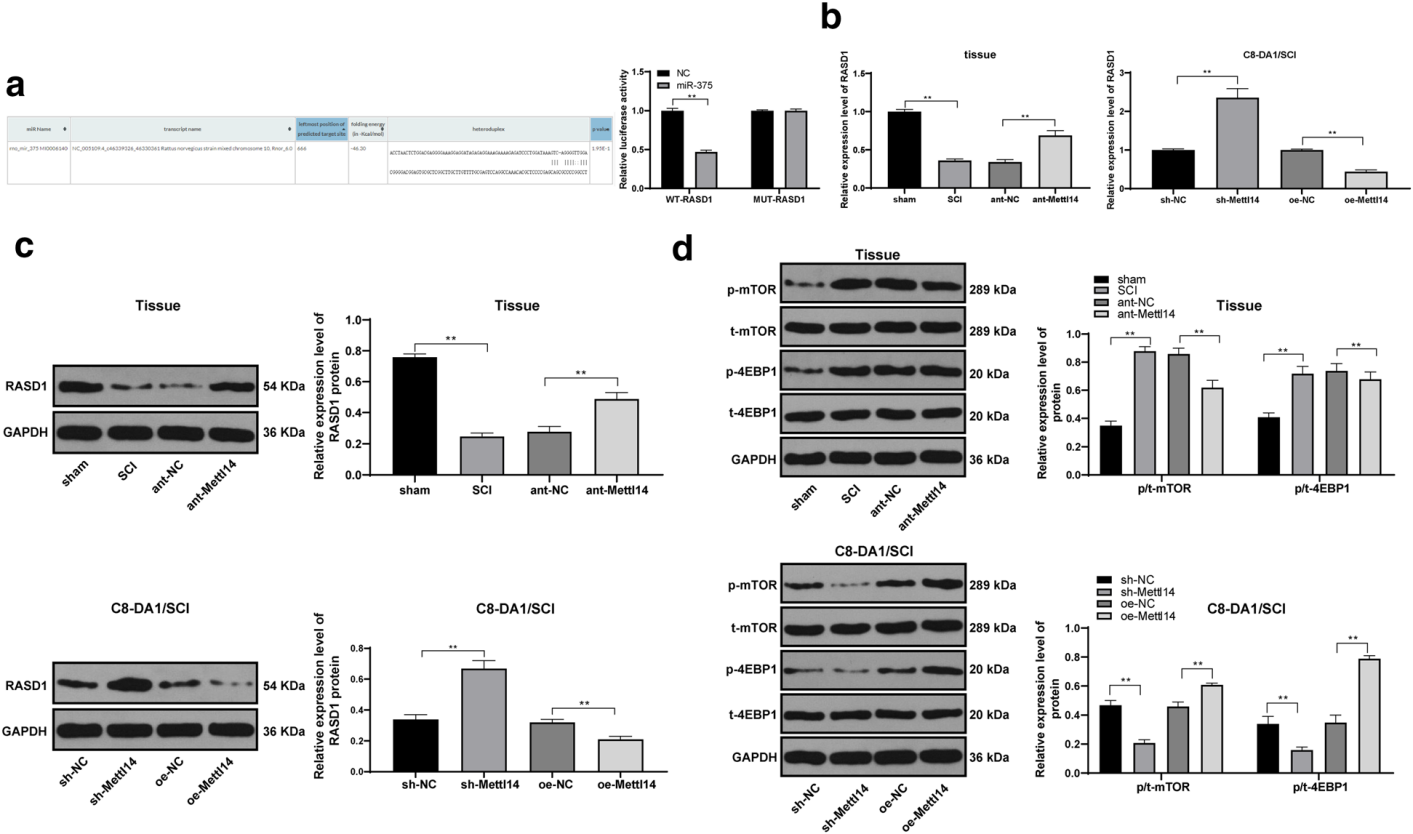
miR-375 targeted RASD1 to activate the mTOR pathway. The target binding relationship between miR-375 and RASD1 was verified by dual-luciferase assay (**a**). Then RT-qPCR (**b**) and Western blot analysis (**c**) were used to detect the level of RASD1 in rat spinal cord and C8-DA1/SCI cells, and Western blot analysis was used to detect the levels of mTOR-related proteins in rat spinal cord and C8-DA1/SCI cells (**d**). Each experiment was repeated three times independently. The data are expressed by mean ± standard deviation. The data in panels B/C were analysed by one-way ANOVA, and data in panels **a**, **d** were analysed by two-way ANOVA, followed by Tukey’s multiple comparisons test. *p < 0.05, **p < 0.01

### miR-375 targeted RASD1 to activate the mTOR pathway

To further understand the downstream mechanism of miR-375, we predicted that there were multiple target genes of miR-375 through the biological website (https://cm.jefferson.edu/rna22/Interactive/) [24], and found a binding site between RASD1 and miR-375. The involvement of RASD1 (also known as Dexras1) in SCI has been reported previously [25]. Therefore, we speculate that miR-375 plays a role in SCI through RASD1. After that, we confirmed a binding site between miR-375 and RASD1 by dual-luciferase experiment (Fig. 6a). RT-qPCR and Western blot analysis indicated that after the silencing Mettl14, RASD1 levels were increased significantly (*p* < 0.05) (Fig. 6b, c). In addition, RASD1 can negatively regulate the Akt/mTOR pathway [26], and inhibition of mTOR pathway has a positive effect on neuronal repair after SCI [27]. Thereafter, we measured mTOR levels in spinal cord tissues and cells. mTOR pathway was inactivated in tissues and cells after treatment with silencing Mettl14 (Fig. 6d). In conclusion, miR-375 targeted RASD1 and activated mTOR pathway in SCI.

### Inhibition of miR-375 or overexpression of RASD1 partially reversed the effect of oe-Mettl14 on the apoptosis of SCI cells 

**Fig. 7:**
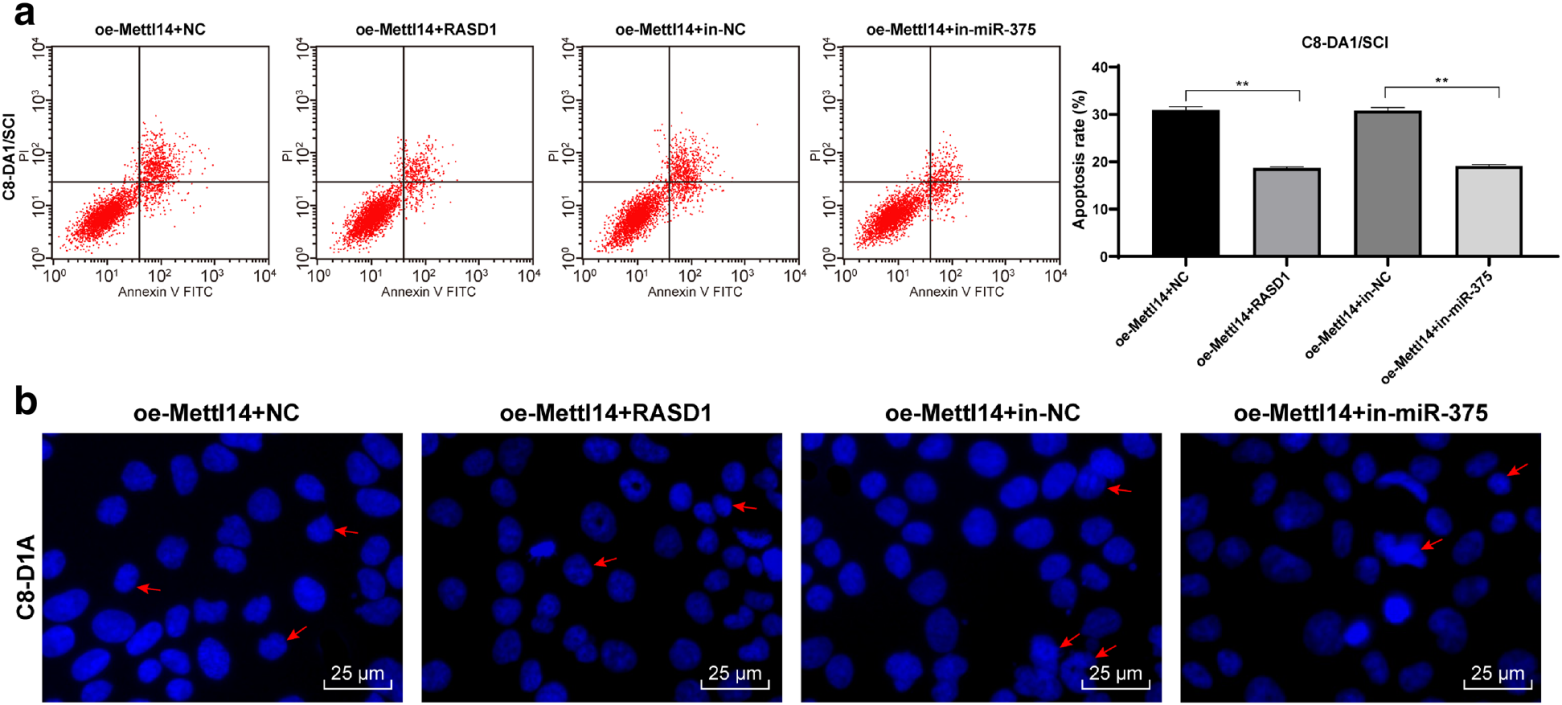
Inhibition of miR-375 or overexpression of RASD1 partially reversed the effect of oe-Mettl14 on the apoptosis of SCI cells. Overexpression of RASD1 and oe-Mettl14, and low expression of miR-375 and oe-Mettl14 were cotransfected into C8-DA1/SCI cells. Apoptosis was evaluated by **a** flow cytometry and **b** Hoechst staining 24 h later. Each experiment was repeated three times independently. The data are expressed by mean ± standard deviation. The data in panels A/B were analysed by the t test. **p < 0.01

These results have confirmed that Mettl14 has a negative effect on SCI by mediating m6A to regulate miR-375 and then inhibit RASD1 expression. To further confirm this conclusion, we conducted functional rescue experiments, to cotransfect overexpression RASD1 and oe-Mettl14, and inhibiting miR-375 and oe-Mettl14 into C8-DA1/SCI cells. Compared with oe-Mettl14 + NC group, the apoptosis of combined groups was inhibited (Fig. 7a, b). In conclusion, overexpression of RASD1 or inhibition of miR-375 can partially reverse the effect of oe-Mettl14 on the apoptosis of SCI cells.

## Discussion

**Fig. 8:**
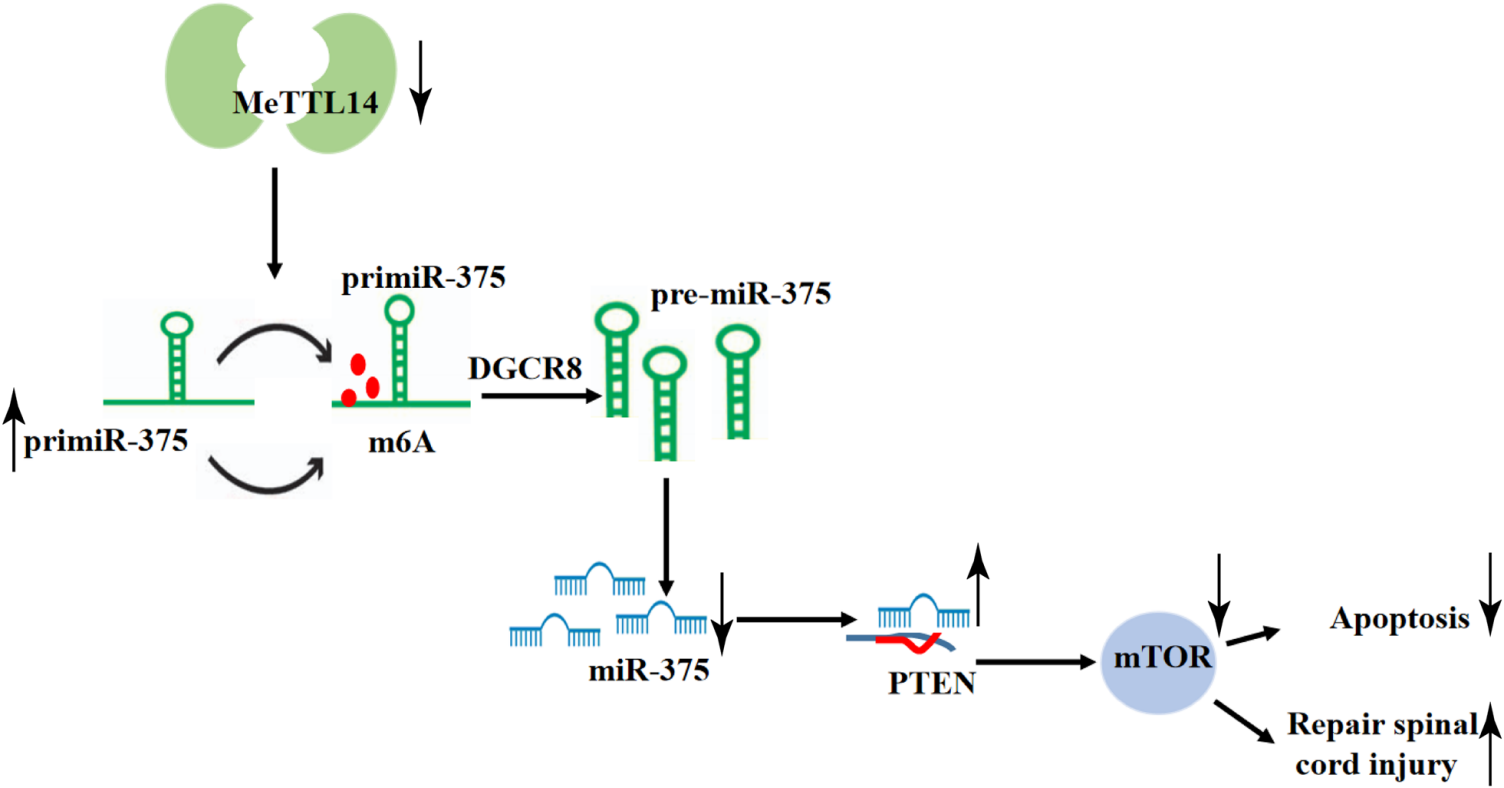
Mechanism chart. Inhibition of Mettl14 expression can inhibit Mettl14-mediated m6A modification by regulating the transformation process from pri-miR-375 to mature pri-miR-375, thus promoting the expression of RASD1, inhibiting the apoptosis of spinal cord neurons and promoting the repair of spinal cord injury

Mettl14 level and m6A content in spinal cord tissue were significantly increased, and Mettl14-mediated m6A modification inhibited RASD1 and promoted neuron apoptosis during SCI repair by promoting the transformation of pri-miR-375 to mature miR-375. These findings revealed a novel mechanism for modulation of apoptosis of spinal cord neurons by Mettl14-mediated m6A modification in the spinal cord (Fig. 8).

m6A modification is critical for neuronal processes and disorders, and learning and memory [28, 29]. Mettl14 and m6A levels were upregulated in breast cancer tissues [12]. In this study, the content of m6A in total RNA and Mettl14 levels of SCI rats were significantly increased. Similarly, level of spinal m6A modification was clearly increased in a mouse model of chronic inflammatory pain, with the augmented Mettl3 in the spinal cord [29]. Given that levels of Mettl14 and m6A are increased in the spinal cord and that Mettl14 is important in mediating m6A formation, we considered whether intervention of Mettl14 could affect SCI in a m6A modification-dependent manner. We silenced Mettl14 expression in the spinal region to assess locomotion recovery in SCI rats. By RT-qPCR screening and gain- or loss-of-function analyses, we identified the motor function of rats was restored after ant-Mettl14 injection, and the damage of central gray matter and peripheral white matter was weakened after ant-Mettl14 injection. Mettl14 deletion reduced m6A methylation, increased neuronal excitability, and impaired striatal-mediated gross motor performance and learning [30]. After deletion of m6A by Mettl14 gene in mouse embryonic brain, the cell cycle of radial glial cells was prolonged, and the cortical neurogenesis was prolonged to postnatal [31]. To our knowledge, the current study is the first one investigating both the expression profiling and mechanism of m6A modification and Mettl14 in SCI models.

Considering the protective effect of Mettl14 deletion in locomotion recovery, we then explore the effect of Mettl14 on neurite recovery. Reduction of AcTub and MAP2 after SCI directly contributed to neuronal dysfunction and death [32]. After ant-Mettl14 injection, AcTub and MAP2 in the spinal cord increased significantly, GFAP and NF-200 expression decreased, while that of NeuN increased significantly. Valproic acid administration promoted MAP2 expression and thus stimulated neurite outgrowth in the SCI model [33]. The number of neurons was evaluated by NeuN-positive cells and astrocytes were by GFAP-positive cells, and axonal growth was assessed by NF-200; the number of NeuN-positive cells was significantly reduced in the SCI while GFAP- and NF-200-positive cells were increased [34]. High-level apoptosis induced by SCI evokes serious damage because of the loss and dysfunction of motor neurons [35]. After ant-Mettl14 injection, TUNEL-positive cells and NeuN + caspase-3-positive cells in the spinal cord were decreased, and neurons survived increased significantly. Consistently, Mettl14 downregulation significantly reduced the apoptosis of H_2_O_2_-induced C8-DA1 and C8-B4 cells. In conclusion, Mettl14 knockdown can promote the recovery of neurites and prevent neuron apoptosis after SCI.

Then our focus shifted to investigating the mechanism of Mettl14-mediated m6A modification. m6A can affect the disease progression by affecting pri-miR transformation to mature miR [22]. Highly upregulated miR-375-3p was observed in the spinal cord of rats with contusive spinal cord injury and treated with oligodendrocyte precursor cell transplantation and miR-375 acted primarily to inhibit cell proliferation of oligodendrocyte precursor cells [23]. Therefore we hypothesized miR-375 was involved in the Mettl14-mediated m6A modification in SCI. In this study, our results supported that miR-375 decreased and pri-miR-375 increased significantly after silencing Mettl14, the mutation of m6A site in pri-miR-375 reduced the processing of mature body, and overexpression of Mettl14 promoted the combination of DGCR8 and pri-miR-375. In addition, RIP assay showed that overexpression of Mettl14 significantly increased the expression of m6A-modified pri-miR-375. To sum up, Mettl14 depletion decreased m6A level, thus restored the stability of pri-miR-375 reduced by m6A modification, and blocked the transformation from pri-miR-375 to miR-375.

Furthermore, we explored the downstream mechanism of miR-375. We confirmed a binding site between miR-375 and RASD1. After silencing Mettl14 expression, RASD1 levels increased significantly. RASD1 was mainly located in neurons in the gray matter [36]. RASD1 may be involved in the different pathological conditions including nerve regeneration, neuron loss or survival and even pain process, possibly via regulating the nNOS activity or through the downstream targets [37]. Overexpression of RASD1 inhibited glioma expansion and inactivated the AKT/mTOR pathway [26]. mTOR pathway is crucial in the functional recovery of central nervous system trauma, especially for axon regeneration and apoptosis in the treatment of SCI [38]. mTOR pathway was inactivated in tissues and cells after Mettl14 knockdown. Resveratrol-mediated inhibition of mTOR pathway may be beneficial for neural recovery after SCI [27]. In conclusion, miR-375 targeted RASD1 and activated mTOR pathway in SCI. To further confirm this conclusion, we conducted functional rescue experiments to co-transfect overexpression RASD1 and oe-Mettl14, and lowly expressed miR-375 and oe-Mettl14 into C8-DA1/SCI cells. Compared with oe-Mettl14 + NC group, the apoptosis of combined groups was inhibited. In conclusion, overexpression of RASD1 or inhibition of miR-375 can partially reverse the effect of oe-Mettl14 on the apoptosis of SCI cells. In the study from Yang et al. [23], oligodendrocyte precursor cell transplantation may promote functional recovery of rats with contusive spinal cord injury, which may be associated with upregulated miR-375-3p. In their study, although the change in miR-375-3p was observed, its effect was not determined. The different treatment method or pathway may cause the discrepancy. We will further make experiments to validate the role of miR-375 in SCI.

## Conclusions

In summary, we unveiled a novel promising approach for SCI treatment by inhibiting the Mettl14-mediated m6A modification through the pri-miR-375 processing. The present study determined the effect of the miR-375 and further studies are warranted to explore the roles of other miRs in SCI.

## Methods

### Establishment of SCI rat model

Sprague Dawley rats of specific pathogen-free grade (all male, 8 weeks old, 200 ± 20 g) supplied by the animal experimental center of Suzhou University were raised at 25 ± 0.5 °C under natural light with free water and food. According to the reference [39], an incision was made in the skin along the medial dorsal line to the aponeurotic and muscular planes, and the posterior vertebral arches were exposed from T8 to T12. Under the dissection stereomicroscope, 3-mm-long laminectomy was performed on the caudal end of T10 vertebra and the rostral end of T11 vertebra. The Infinite Horizons impactor (Infinite Horizons, L.L.C., Lexington, KY, USA) was adopted to produce the contusion SCI using a force of 60 kdyn/cm^2^. The SCI model rats were established and randomly assigned to SCI model group, ant-NC (negative control, SCI rats treated with lentiviral (lv)-shRNA NC of Mettl14) group and ant-Mettl14 group (SCI rats treated with lv-shRNA of Mettl14). Rats were subjected to laminectomy and then treated with lv-shRNA Mettl14/lv-shRNA-NC (50 µL/day, 100 nmoL/mL; RiboBio, Guangzhou, China) via an intrathecal injection through lumbar puncture [40] for 3 days (0, 1, and 2 days) after 15 min of SCI modelling. In addition, the unmodeled rats were set as sham group.

### Assessment of locomotion recovery

The behaviors of the rats were tested at 0, 5, 10, 15, 20, 25, and 30 days after operation. BBB scores and inclined plane test were utilized to evaluate the locomotion recovery in SCI rats [41]. BBB scores ranged from 0 point (complete paralysis) to 21 points (normal locomotion) based on the muscle strength and joint movement. Rats were examined in right side or left side up. The time for a rat keeping one position for 5 s without falling was recorded. These examinations were performed by two independent researchers after surgery.

### Histological experiment

All rats were euthanized by an intraperitoneal injection of 800 mg/kg pentobarbital on the 30th day after operation. Eight rats in each group were selected. The injured spinal cord tissue was taken as the injury center. The spinal cord tissue with a length of 2 cm was cut and embedded in paraffin. The following histological experiments were carried out after equal distance transverse sectioning. The transverse sections of comparable distance to the injured site at 5 mm thickness were used for hematoxylin and eosin (HE) staining (Beijing Solarbio Science & Technology Co., Ltd., Beijing, China). Transverse sections of comparable distance to the injured site were treated with 1% cresyl violet acetate for Nissl staining (Solarbio) to evaluate surviving neurons. The spinal cord tissues in the same site of other rats in each group were prepared into homogenate for RT-qPCR and Western blot assay. The distance between the sampling region and the injured site was equal in different groups.

### Quantification of m6A RNA methylation

Total RNA was extracted from tissues using TRIzol (Invitrogen, Carlsbad, CA, USA), and the quality was determined using NanoDrop (Thermo Fisher Scientific, Waltham, MA, USA) and 1% agarose gel electrophoresis. The m6A content in high-quality RNA was examined using EpiQuik™ m6A RNA methylation quantification kit (P-9005-48, Colorimetric, Epigentek, USA). In brief, 100–300 ng RNA was loaded into the wells, and capture and detection antibody solutions were separately put into each well. Afterwards, m6A levels were detected based on the absorbance at 450 nm, and relative m6A content was calculated using a standard curve.

### Reverse transcription quantitative polymerase chain reaction (RT-qPCR)

Total RNA was extracted from spinal cord segments and cells by TRIzol (Invitrogen), and reverse transcribed to cDNA using Prime-Script RT reagent kit (Takara, Tokyo, Japan). SYBR Premix Ex Taq (Takara) and primers (Table 1) (Ribobio) were added to cDNA mixtures for RT-qPCR. The relative mRNA expression was normalized to GAPDH.

**Table 1:** Primer sequences

Gene	Sequence (5′-3′)
Mettl14	F: TTTCTCTGGTGTGGTTCTGG
	R: AAGTCTTAGTCTTCCCAGGATTG
U6	F: CTCGCTTCGGCAGCACA
	R: GTGTCGTGGAGTCGGCAA
miR-375	F: AAGCTTTGTTCGTTCGGCTC
	R: GTATCCAGTGCGAATACCTC
RASD1	F: TGAGAAAAATGCCAAGCGCC
	R: GGAGTCTTGAGGGGAGTGGA
GAPDH	F: ACCAGGTATCTGCTG GTTG
	R: TAACCATGATGTCAGCGTGGT

### Western blot assay

Spinal cord tissue and cells were lysed by RIPA with phosphatase inhibitors and protease inhibitors cocktail, and protein concentration was determined using a bicinchoninic acid reagent (ThermoFisher). Equal amount of proteins was separated using 8–12% SDS-PAGE gels, and transferred to polyvinylidene fluoride membranes (Bio-Rad, Hercules, CA, USA). Then the membranes were blocked with 5% nonfat milk and incubated with primary antibodies: Mettl14 (1:1000, #51,104, CST, Beverly, MA, USA), MAP2 (1 µg/mL, ab11267, Abcam), acetyl-α-tubulin (0.06 µg/mL, ab24610, Abcam), Iba-1 (1:500, ab178846, Abcam), RASD1 (0.4 µg/mL, NBP1-91830, Shanghai Univ Biotechnology Co., Ltd., Shanghai, China), p-4EBP1 (1:1000, #2855, CST), t-4EBP1 (1:5000, ab32024, Abcam), p-mTOR (1:1000, ab109268, Abcam), t-mTOR (1:1000, ab32028, Abcam), and secondary antibodies (1:2000, ab205718 or 1:2000, ab205719; Abcam). The bands were examined by the ChemiDic XRS + Imaging System (Bio-Rad), and band intensities were analyzed with Image Lab 3.0 software (Bio-Rad). Experiments were performed at least three times.

### Immunohistochemical staining

Sections were added with an auto-fluorescent quencher to prevent auto-fluorescence. Then, the sections were mounted with 10% normal serum blocking solution, which was the same species as the secondary antibody to avoid unspecific staining. After that, the sections were incubated with primary antibodies Mettl14 (1:1000, ab220030, Abcam) and neuronal nuclei (NeuN) (1:500, ab177487, Abcam) at 4 ℃ overnight. After phosphate-buffered saline (PBS) washing, sections were cultured for 50 min with secondary antibody Alexa Fluor 488-conjugated goat anti-mouse immunoglobulin G (IgG) (1:1000, ab150117, Abcam). The nuclei were stained with 4′,6-diamidino-2-phenylindole (1:500, ab104139, Abcam). The stained sections and the lentiviral vector-induced green auto-fluorescence were visualized using a fluorescence microscope (Nikon, Tokyo, Japan).

### TUNEL assay

TUNEL assay was conducted using the fluorescent TUNEL kit (Roche, Basel, Switzerland) based on FITC fluorescein labeling and positive apoptotic nuclei by fluorescence microscopy. The sections were immersed in cold acetone for 10 min. After PBS (pH = 7.4) washing, the sections were cultured at 37 ℃ for 25 min with proteinase K solution and broken membrane solution for 20 min. Following PBS washing, sections were immersed for 2 h at 37 ℃ in the mixture of reagent 1 (TdT) and reagent 2 (dUTP) at 1:9. After that, the nuclei were stained for 10 min with DAPI. Images were obtained and positive cells were counted and analyzed using ImageJ software (1.51, NIH).

### Establishment of SCI cell model

Murine astrocytes C8-D1A (Cat No. CRL-2541) and C8-B4 (Cat No. CRL-2540) purchased from ATCC were cultured in Dulbecco’s modified Eagle’s medium (DMEM, ATCC, Manassas, VA, USA) with 10% (v/v) fetal bovine serum (Invitrogen) at 37 ℃ in a humidified atmosphere containing 5% CO_2_. The media were renewed every 3 day. Cells were passaged at 1:4 when growing to 80% confluency. The apoptosis was induced in SCI model using hydrogen peroxide [42]. According to the literature report [43], hydrogen peroxide (Sigma-Aldrich, Merck KGaA, Darmstadt, Germany) at 10 µM was added into C8-D1A/C8-B4 cells for 12 h.

### Cell transfection

When C8-D1A/C8-B4 cells reached 80% confluency in the 6-well plates, cells were transfected with 2 µg shRNA of Mettl14 (sh-Mettl14) or its shRNA NC (sh-NC), overexpression plasmid of Mettl14 (oe-Mettl14) or its NC (oe-NC), RASD1 pcDNA (RASD1) or its NC (NC), miR-375 inhibitor (in-miR-375) or its inhibitor NC (in-NC) (all constructed and synthesized by Ribobio) at 37 ℃ for 48 h using Lipofectamine® 2000 (Invitrogen). After 24 h, following experiments were carried out.

### Flow cytometry

The spinal cord tissues of rats were cut, suspended, and washed in PBS, and centrifuged for 2 min at 1000 r/min after filtration. After discarding the supernatant, the sections were added with 5 µL annexin V-FITC, incubated in the dark for 15 min, then added with 2.5 µL PI staining (A1003-12-5, Nanjing JianCheng Bioengineering Institute, Nanjing, Jiangsu, China) solution and mixed well. The neuron apoptosis was measured by flow cytometry.

### Hoechst staining

The cells in logarithmic growth phase were detached, centrifuged, resuspended and counted. The cells were adjusted into appropriate concentration and seeded into 24-well plates. Each well was added with 500 µL cell suspension. The cells were cultured in an incubator at 37 ℃ with 5% CO_2_ and relative humidity for 24 h. After that, 5 µL of Hoechst 33,342 living cell staining solution (Beijing baiaoleibo) was evenly added into the culture medium and mixed gently. The cells were incubated for 10 min. The waste liquid was washed twice with PBS, and the apoptosis was observed under fluorescence microscope.

### Dual‐luciferase reporter gene assay

Cells were cotransfected with plasmids containing 3′-UTR of wild or mutant fragments from RASD1 and miR mimic using Lipofectamine 3000 (Invitrogen). After transfection for 24 h, firefly and renilla luciferase activities were assessed using dual-luciferase system (Promega, Madison, Wisconsin, USA). Finally, the ratio of luminescence from firefly to renilla luciferase was calculated.

### Ectopic reporter constructs

The primiRNA reporter construct developed by Auyeung et al. [44] was employed, and the miR control pri-miR-1-1 was replaced with its altered version, in which the adenosines (A’s) of the potential m6A motifs were mutated. Then we placed the query miR, either WT pri-miR-375 or a mutant version in which the A’s of the putative m6A motifs were mutated to thymidines (Ts), upstream of the pri-miR-1-1 control. Then these two constructs were used to transfect HEK293T cells using Lipofectamine 2000. The RNA was extracted 48 h later, and RT-qPCR was performed to evaluate the production of mature miR-375 and mature miR-1-1.

### RNA immunoprecipitation (RIP) assay

A RIP assay was performed with a Magna RIP RNA-Binding Protein Immunoprecipitation Kit (Millipore, Billerica, MA, USA) in accordance with the manufacturer’s protocol. Cells stably transfected with Mettl4 overexpression plasmids (oe-Mettl14) or control (oe-NC) were lysed with RIP lysis buffer. Immunoprecipitation of endogenous DGCR8 was conducted using an anti-DGCR8 antibody (1:1000, ab191875, Abcam) overnight at 4 ℃. After washes, the immunoprecipitated protein-RNA complex was analyzed and treated with Proteinase K. RNAs were extracted and subjected to RT-qPCR for pri-miR-375.

For the m6A RNA binding assays, the Magna MeRIP m6A Kit (Millipore) was used. RNAs were fragmented and incubated with magnetic beads conjugated with m6A antibody (Millipore) for immunoprecipitation. The enrichment of pri-miR-375 was analyzed using RT-qPCR.

### Statistical analysis

Statistical analysis was conducted by SPSS21.0 (IBM Corp. Armonk, NY, USA). The Kolmogorov-Smirnov test checked whether the data were normally distributed. The measurement data are presented as the mean ± standard deviation. The *t* test was applied for comparisons between two groups, and one-way analysis of variance (ANOVA) or two-way ANOVA was used for multi-groups, with Tukey’s multiple comparisons test for pair-wise comparisons after ANOVA analyses. The Pearson test was used for correlation analysis. The *p* value was obtained by two-tailed tests, and *p* < 0.05 indicated significant differences.

.

## Data Availability

The data that support the findings of this study are available from the corresponding author upon reasonable request.

## Abbreviations

SCI: Spinal cord injury
Mettl14: Methyltransferase-like 14
m6A: N6‑methyladenosine
miR: MicroRNA
NC: Negative control
BBB: Beattie and Bresnahan
HE: Hematoxylin and eosin
ELISA: Enzyme-linked immunosorbent assay
IL: Interleukin
TNF-α: Tumor necrosis factor-α
RT-qPCR: Reverse transcription quantitative polymerase chain reaction
PBS: Phosphate-buffered saline
MAP2: Microtubule associated protein 2
IgG: Immunoglobulin G
DMEM: Dulbecco’s modified Eagle’s medium
OE: Overexpression
RIP: RNA immunoprecipitation
ANOVA: Analysis of variance

## References

[1] Ray SK (2020). Modulation of autophagy for neuroprotection and functional recovery in traumatic spinal cord injury. Neural Regen Res.

[2] Tang R, Botchway BOA, Meng Y (2020). The inhibition of inflammatory signaling pathway by secretory leukocyte protease inhibitor can improve spinal cord injury. Cell Mol Neurobiol.

[3] Chiodo AE, Sitrin RG, Bauman KA (2016). Sleep disordered breathing in spinal cord injury: a systematic review. J Spinal Cord Med.

[4] Kornhaber R, McLean L, Betihavas V (2018). Resilience and the rehabilitation of adult spinal cord injury survivors: a qualitative systematic review. J Adv Nurs.

[5] Hamid R, Averbeck MA, Chiang H (2018). Epidemiology and pathophysiology of neurogenic bladder after spinal cord injury. World J Urol.

[6] Yamazaki K, Kawabori M, Seki T (2020). Clinical trials of stem cell treatment for spinal cord injury. Int J Mol Sci.

[7] Ren XD, Wan CX, Niu YL (2019). Overexpression of lncRNA TCTN2 protects neurons from apoptosis by enhancing cell autophagy in spinal cord injury. FEBS Open Bio.

[8] Shao Z, Lv G, Wen P (2018). Silencing of PHLPP1 promotes neuronal apoptosis and inhibits functional recovery after spinal cord injury in mice. Life Sci.

[9] Xiang M, Liu W, Tian W (2020). RNA N-6-methyladenosine enzymes and resistance of cancer cells to chemotherapy and radiotherapy. Epigenomics.

[10] Frye M, Harada BT, Behm M (2018). RNA modifications modulate gene expression during development. Science.

[11] Machnicka MA, Milanowska K, Osman Oglou O (2013). MODOMICS: a database of RNA modification pathways–2013 update. Nucleic Acids Res.

[12] Yi D, Wang R, Shi X (2020). METTL14 promotes the migration and invasion of breast cancer cells by modulating N6methyladenosine and hsamiR146a5p expression. Oncol Rep.

[13] Huang J, Dong X, Gong Z (2019). Solution structure of the RNA recognition domain of METTL3-METTL14 N(6)-methyladenosine methyltransferase. Protein Cell.

[14] Ma JZ, Yang F, Zhou CC (2017). METTL14 suppresses the metastatic potential of hepatocellular carcinoma by modulating N(6) -methyladenosine-dependent primary MicroRNA processing. Hepatology.

[15] Lee M, Kim B, Kim VN (2014). Emerging roles of RNA modification: m(6)A and U-tail. Cell.

[16] Ghibaudi M, Boido M, Vercelli A (2017). Functional integration of complex miRNA networks in central and peripheral lesion and axonal regeneration. Prog Neurobiol.

[17] Shi Z, Zhou H, Lu L (2017). The roles of microRNAs in spinal cord injury. Int J Neurosci.

[18] Wang P, Doxtader KA, Nam Y (2016). Structural Basis for Cooperative Function of Mettl3 and Mettl14 Methyltransferases. Mol Cell.

[19] Yu F, Li P, Du S (2020). Olfactory ensheathing cells seeded decellularized scaffold promotes axonal regeneration in spinal cord injury rats. J Biomed Mater Res A.

[20] Zhan Z, Wu Y, Liu Z (2020). Reduced dendritic spines in the visual cortex contralateral to the optic nerve crush eye in adult mice. Invest Ophthalmol Vis Sci.

[21] Zhong ZX, Feng SS, Chen SZ (2019). Inhibition of MSK1 Promotes Inflammation and Apoptosis and Inhibits Functional Recovery After Spinal Cord Injury. J Mol Neurosci.

[22] Han J, Wang JZ, Yang X (2019). METTL3 promote tumor proliferation of bladder cancer by accelerating pri-miR221/222 maturation in m6A-dependent manner. Mol Cancer.

[23] Yang J, Xiong LL, Wang YC (2018). Oligodendrocyte precursor cell transplantation promotes functional recovery following contusive spinal cord injury in rats and is associated with altered microRNA expression. Mol Med Rep.

[24] Miranda KC, Huynh T, Tay Y (2006). A pattern-based method for the identification of MicroRNA binding sites and their corresponding heteroduplexes. Cell.

[25] Li X, Cheng C, Fei M (2008). Spatiotemporal expression of Dexras1 after spinal cord transection in rats. Cell Mol Neurobiol.

[26] Gao S, Jin L, Liu G (2017). Overexpression of RASD1 inhibits glioma cell migration/invasion and inactivates the AKT/mTOR signaling pathway. Sci Rep.

[27] Zhou J, Huo X, Botchway BOA (2018). Beneficial effects of resveratrol-mediated inhibition of the mTOR pathway in spinal cord injury. Neural Plast..

[28] Satterlee JS, Basanta-Sanchez M, Blanco S (2014). Novel RNA modifications in the nervous system: form and function. J Neurosci.

[29] Zhang C, Wang Y, Peng Y (2020). METTL3 regulates inflammatory pain by modulating m(6)A-dependent pri-miR-365-3p processing. FASEB J.

[30] Koranda JL, Dore L, Shi H (2018). Mettl14 is essential for epitranscriptomic regulation of striatal function and learning. Neuron.

[31] Yoon KJ, Ringeling FR, Vissers C (2017). Temporal Control of Mammalian Cortical Neurogenesis by m(6)A Methylation. Cell.

[32] Chen J, Wang Z, Zheng Z (2017). Neuron and microglia/macrophage-derived FGF10 activate neuronal FGFR2/PI3K/Akt signaling and inhibit microglia/macrophages TLR4/NF-kappaB-dependent neuroinflammation to improve functional recovery after spinal cord injury. Cell Death Dis.

[33] Abdanipour A, Schluesener HJ, Tiraihi T (2015). Systemic administration of valproic acid stimulates overexpression of microtubule-associated protein 2 in the spinal cord injury model to promote neurite outgrowth. Neurol Res.

[34] Wang J, Li H, Chen L (2020). mRNA profiling for miR-124-mediated repair in spinal cord injury. Neuroscience.

[35] Li Y, Lin S, Xu C (2018). Triggering of Autophagy by Baicalein in Response to Apoptosis after Spinal Cord Injury: Possible Involvement of the PI3K Activation. Biol Pharm Bull.

[36] Gao H, Gao Y, Li X (2010). Spatiotemporal patterns of dexamethasone-induced Ras protein 1 expression in the central nervous system of rats with experimental autoimmune encephalomyelitis. J Mol Neurosci.

[37] Shen A, Chen M, Niu S (2008). Changes in mRNA for CAPON and Dexras1 in adult rat following sciatic nerve transection. J Chem Neuroanat.

[38] Lin J, Huo X, Liu X (2017). “mTOR Signaling Pathway”: a potential target of curcumin in the treatment of spinal cord injury. Biomed Res Int..

[39] Wan G, An Y, Tao J (2020). MicroRNA-129-5p alleviates spinal cord injury in mice via suppressing the apoptosis and inflammatory response through HMGB1/TLR4/NF-kappaB pathway. Biosci Rep.

[40] Shu B, He SQ, Guan Y (2020). Spinal cord stimulation enhances microglial activation in the spinal cord of nerve-injured rats. Neurosci Bull.

[41] Zhang HY, Wang ZG, Wu FZ (2013). Regulation of autophagy and ubiquitinated protein accumulation by bFGF promotes functional recovery and neural protection in a rat model of spinal cord injury. Mol Neurobiol.

[42] Wu WD, Wang LH, Wei NX (2019). MicroRNA-15a inhibits inflammatory response and apoptosis after spinal cord injury via targeting STAT3. Eur Rev Med Pharmacol Sci.

[43] Campbell AM, Zagon IS, McLaughlin PJ (2013). Astrocyte proliferation is regulated by the OGF-OGFr axis in vitro and in experimental autoimmune encephalomyelitis. Brain Res Bull.

[44] Auyeung VC, Ulitsky,McGeary ISE (2013). Beyond secondary structure: primary-sequence determinants license pri-miRNA hairpins for processing. Cell.

