# Correction to: Mettl14-mediated m6A modification modulates neuron apoptosis during the repair of spinal cord injury by regulating the transformation from pri‐mir‐375 to miR-375

**DOI:** 10.1186/s13578-021-00573-w

**Published:** 2021-04-02

**Authors:** Haoyu Wang, Jing Yuan, Xiaoqian Dang, Zhibin Shi, Wenrui Ban, Dong Ma

**Affiliations:** 1 Department of Orthopedics, Xi’an Jiaotong University Second Affiliated Hospital, Xi’an, 710004 Shanxi People’s Republic of China; 2 Xi’an Radio and Television University, Xi’an, 710002 Shanxi People’s Republic of China; 3 Key Laboratory of Shanxi Province for Craniofacial Precision Medicine Research, College of Stomatology, Xi’an Jiaotong University, 98 XiWu Road, Xi’an, 710004 Shaanxi China

## Correction to: Cell Biosci (2021) 11:52 10.1186/s13578-020-00526-9

Following publication of the original article [1], the authors identified an error in Fig. 8. The correct figure is given below.

**Fig. 8:**
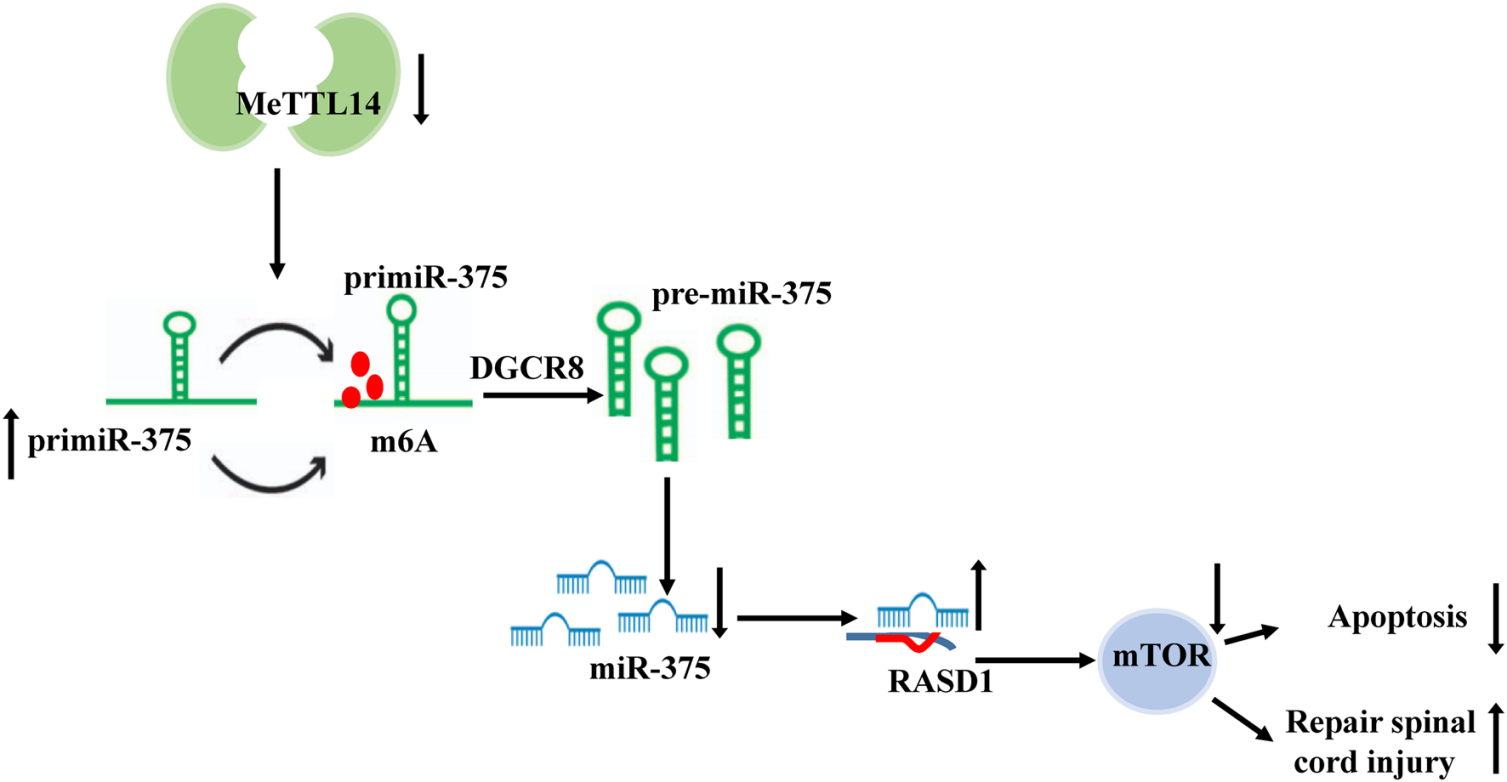
Mechanism chart. Inhibition of Mettl14 expression can inhibit Mettl14-mediated m6A modification by regulating the transformation process from pri-miR-375 to mature pri-miR-375, thus promoting the expression of RASD1, inhibiting the apoptosis of spinal cord neurons and promoting the repair of spinal cord injury

The original article has been corrected.
